# Significant liver histological change is common in HBeAg-positive chronic hepatitis B with normal ALT

**DOI:** 10.1186/s12879-024-09617-1

**Published:** 2024-07-23

**Authors:** Menghui Duan, Huanming Xiao, Meijie Shi, Yubao Xie, Pengtao Zhao, Sheng Li, Xiaoling Chi, Xueen Liu, Hui Zhuang

**Affiliations:** 1 Department of Microbiology and Center of Infectious Diseases, School of Basic Medical Sciences, Peking University Health Science Center, 38 Xueyuan Road, Haidian District, Beijing, 100191 China; 2 Hepatology Department, Guangdong Provincial Hospital of Chinese Medicine, 111 Dade Road, Guangzhou, Guangdong Province 510120 China; 3 The Clinical Laboratory of Tianjin Chest Hospital, Tianjin, China

**Keywords:** ALT, normal, Chronic hepatitis B, HBV DNA, Immune tolerance, Liver histology

## Abstract

**Background and aims:**

Numerous HBeAg-positive chronic hepatitis B (CHB) patients with persistently normal ALT have significant liver histopathology. It is imperative to identify true “immune tolerant” patients. We aimed to evaluate the liver histopathology features of HBeAg-positive CHB patients with normal ALT and the incidence of liver cirrhosis and HCC in CHB patients during follow-up.

**Methods:**

179 HBeAg-positive CHB patients with normal ALT who performed liver biopsy from 2009 to 2018 were retrospectively analyzed. Liver necroinflammation ≥ G2 and/or liver fibrosis ≥ S2 was defined as significant liver histopathological change.

**Results:**

57.5% patients were in the indeterminate phase with significant liver histological changes. The proportion of the patients with evident liver necroinflammation was higher in the high-normal ALT group (21-40U/L) when compared with the low-normal ALT group (≤ 20 U/L) (51.3% vs. 30.0%, *p* < 0.05), and patients aged ≥ 40 years had a higher proportion of significant fibrosis than those aged < 40 years (64.5% vs. 39.9%, *p* < 0.05). The percentages of patients with ≥ S2 and ≥ G2/S2 in the HBV DNA < 10^7^ IU/mL group were higher than those in the HBV DNA ≥ 10^7^ IU/mL group (72.7% vs. 40.1%, *p* < 0.01; 81.8% vs. 54.1%, *p* < 0.05). During follow-up, two of immune tolerant patients and four of indeterminate patients developed into cirrhosis, and one of immune tolerant patients and one of indeterminate patients developed into HCC, respectively.

**Conclusions:**

HBeAg-positive CHB patients with high-normal ALT or HBV DNA < 10^7^ IU/mL were tend to be indeterminate. Liver biopsy or noninvasive approaches are recommended to evaluate liver histopathology, and antiviral therapy is recommended for patients with significant liver histopathology.

**Supplementary Information:**

The online version contains supplementary material available at 10.1186/s12879-024-09617-1.

## Introduction

According to the study on the global prevalence for chronic hepatitis B virus (HBV) infection, approximately 292 million people worldwide are affected, with nearly 59.4 million patients being immune-tolerant. Morever, about 86 million people are affected in China, including 15.84 million patients in the immune tolerant phase [1]. Chronic HBV infection is still a major public health concern.

Chronic HBV infection is usually classified as immune tolerant, immune clearance, inactive carrier, and reactive phase [2]. HBeAg-positive CHB patients with high levels of HBV DNA and persistent normal ALT are usually clinically diagnosed as “immune tolerant” patients, and the risk of disease progression is minimal. The main international guidelines have diverse definitions of the immune tolerant phase. According to the American Association for the Study of the Liver Disease (AASLD) guidelines, the upper limit of normal ALT (ULN) is 35 U/L for men and 25 U/L for women, [3] while the European Association for the Study of the Liver (EASL) and the Asian Pacific Association for the Study of the Liver (APASL) guidelines suggest 40 U/L for both men and women [4, 5]. Additionally, some guidelines propose adopting a more stringent ALT cutoff value: 30 U/L for men and 19 U/L for women [6–8]. Besides persistently normal ALT, the cutoff value of HBV DNA defined by various international guidelines differs in this phase; APASL defines HBV DNA > 2 × 10^5^ IU/mL (ALT is 1 ~ 2×ULN)^5^, EASL defined it as HBV DNA > 10^7^ IU/mL^4^, and AASLD set it as HBV DNA > 10^6^ IU/mL^3^. This shows that there is no consensus on clinical criteria for defining the immune-tolerant phase. However, this concept has been challenged in recent years. Some recent studies have found that a sizable number of HBeAg-positive CHB patients with persistently normal ALT have significant liver injury [9], suggesting a high risk of liver cirrhosis and HCC, and some studies have demonstrated that HBV-specific T cells, HBV DNA integration and clonal hepatocyte proliferation were the immune tolerance patients’ specific charateristics [10–12]. Therefore, identifying genuine “immune tolerant” patients among HBeAg-positive CHB patients with normal ALT is indispensable, further studies are needed to investigate the definition of true immune tolerant phase.

Thus, we retrospectively analyzed 179 HBeAg-positive CHB patients with normal ALT, aiming to study rates and determinants of clinically significant liver injuries.

## Methods

### Study population

The study enrolled 179 chronic hepatitis B patients who performed liver biopsy in Guangdong Provincial Hosipital of Chinese Medicine from April 2009 to December 2018. The procedure of this study conformed to the ethical standards of the committee responsible for human trials and the ethical standards of the latest edition of the Declaration of Helsinki, which was approved by the Ethics Committee of Guangdong Provincial Hospital of Chinese Medicine (No. BF2018-175-01).

Also, the patient’s informed consents were obtained. The research proposal was registered on http://www.chictr.org.cn ChiCTR1900025897,9/13/2019). Eligible participants were in chronic HBV infection, HBsAg positive for more than 6 months with at least 6 months of normal ALT (≤ ULN, 40 U/L), and had liver biopsy results. Study exclusion criteria were as follows: (1) evidence of co-infection of HIV (human immunodeficiency virus), HCV(hepatitis C virus) or HDV (hepatitis D virus); (2)EBV, CMV, Wilson’s disease or schistosomiasis liver disease; (3) underwent antiviral treatment; (4) significant alcohol consumption (≥ 40 g/d for males and ≥ 20 g/d for females); (5)and cirrhosis and HCC etc.

### Liver biopsy

Liver biopsy and pathological diagnosis were also conducted in Guangdong Provincial Hospital of Chinese Medicine. The patient voluntarily signed the informed consent form and adhered to the protocol requirements. The liver tissue was obtained by percutaneous puncture with a 16G puncture needle, and promptly fixed with 10% formalin, paraffin embedded, serial sectioned, and hematoxylin-eosin (HE) staining and Mason staining followed by pathological analysis. The pathological diagnosis of liver tissue was carried out by experienced pathologists, who were blinded to the clinical and biochemical data of the individual patient.

### Definition

Liver necroinflammation histological grading and liver fibrosis staging were guided by Chinese guidelines for the prevention and treatment of CHB (2022 version). Necroinflammation was divided into G0 ~ G4, while liver fibrosis was S0 ~ S4. Necroinflammatory grade ≥ 2 (≥ G2) and/or fibrosis stage ≥ 2 (≥ S2) were recognized as significant liver histopathological change. Indeterminate patients in this study were defined as ALT normal but significant liver histological changes.

The definitions of the Chinese staging of liver histological inflammation and fibrosis in comparisons with Metavir or Ishak have been presented in Chinese guidelines for the prevention and treatment of CHB (2022 version). For example, the Chinese liver fibrosis staging of S0, S1, S2, S3, and S4 approximately corresponds to the Metavir fibrosis staging of F0 (0 score), F1(1 score), F2 (2 score), F3 (3 score), and F4 (4 score).

### Data collection of laboratory tests

Patients’ clinical data have been extracted from the electronic medical record, including ALT, aspartate aminotransferase (AST) and HBV DNA levels (the ULN of ALT and AST for both males and females was 40 U/L). HBV immune markers, such as HBsAg, anti-HBs, HBeAg, anti-HBe and anti-HBc were also collected.

### Statistical analysis

All statistical analyses were carried out using SPSS ver.26.0. The Mann–Whitney U test was used to analyze the quantitative variables which were non-normally distributed and represented as medians and ranges, while the analysis of categorical variables expressed as frequencies and percentages was performed by chi-square test. Correlations between liver histological changes and clinical factors were tested by using Spearman rank correlation. Multivariate logistic regression was adopted to explore the independent risk factors related to significant liver histological changes. A two-sided *p* value of < 0.05 indicates a statistically significant difference.

## Results

### Patients characteristics

**Fig. 1:**
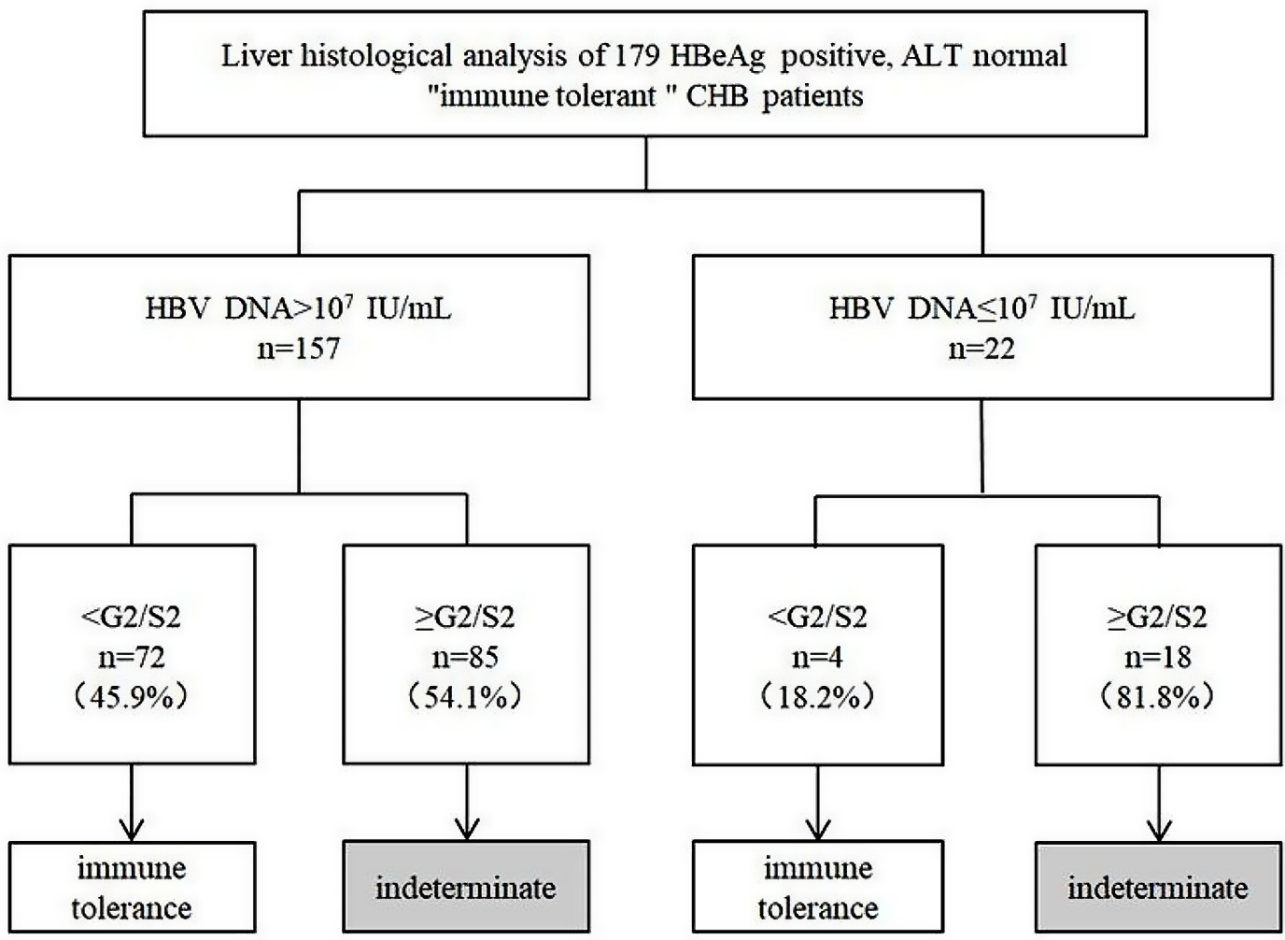
Disease phase distribution of 179 HBeAg-positive “immune tolerant” CHB patients

The patients’ baseline characteristics are described in Table 1. Of the 179 HBeAg-positive patients, the case of patients with low-normal ALT (ALT ≤ 20 U/L) was 60, and that with high-normal ALT (ALT > 20 U/L) was 119. The age of the two groups showed no significant difference. The proportion of males in the group with high normal ALT was significantly higher than that in the group with low normal ALT, and the ALT, AST, and GGT levels were also higher. In addition, the group with high normal ALT had a higher proportion of patients with ≥ G2 (51.3% vs. 30.0%, *p* < 0.05). As shown in Fig. 1 and 103 (57.5%) of patients had significant liver histological changes.

**Table 1:** Baseline characteristics of 179 HBeAg-positive CHB patients

Baseline Characteristics	Total	ALT ≤ 20 U/L	20 < ALT ≤ 40 U/L	*p* value
**Case**	179	60	119	
**Age (years)**	31 (18–58)	30 (22–50)	31(18–58)	0.434
**Male**,** n (%)**	95 (53.1)	23 (38.3)	72 (60.5)	0.005
**ALT (U/L)**	25 (8–40)	16.5 (8–20)	31 (21–40)	0.000
**AST (U/L)**	23 (2–73)	18 (12–40)	25 (2–73)	0.000
**HBV DNA (log IU/mL)**	8.0 (4.2–10.3)	8.1 (4.6–10.3)	7.9 (4.2–9.3)	0.052
**HBsAg (IU/mL)**	1795 (87-10319)	1565 (87-10319)	1880.5 (132–8931)	0.140
**GGT (U/L)**	17 (3.3–121)	15 (5.2–36)	19 (3.3–121)	0.000
**PLT (10** ^**9**^ **/mL)**	200 (73–369)	197.5 (89–333)	200 (73–400)	0.432
**FIB-4**	0.73 (0.03–2.99)	0.71 (0.29–1.67)	0.76 (0.03–2.99)	0.587
**Necroinflammation n (%)**				
**G1**	100 (55.9)	42 (70.0)	58 (48.7)	
**≥G2**	79 (44.1)	18 (30.0)	61 (51.3)	0.007
**Fibrosis n (%)**				
**S0**	16 (8.9)	4 (6.7)	12 (10.1)	
**S1**	84 (46.9)	35 (58.3)	49 (41.2)	
**≥S2**	79 (44.1)	21 (35.0)	58 (48.7)	0.081

### Subgroup comparison of different age

**Fig. 2:**
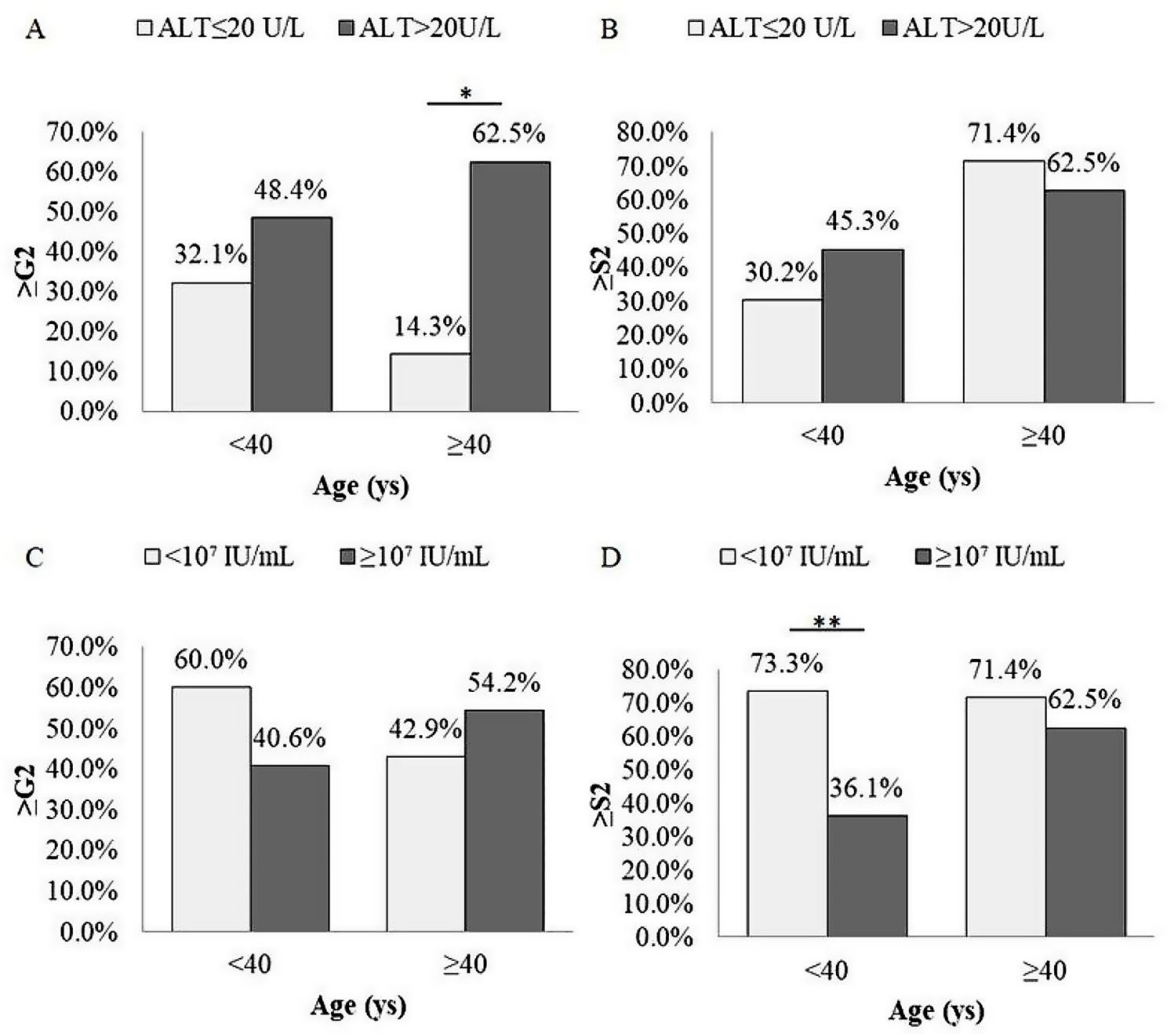
Significant liver histopathology in HBeAg-postive CHB patients in different age and ALT **(A**,** B)** groups; age and HBV DNA groups **(C**,** D).** Statistical analysis was performed by chi-square test, *p < 0.05, **p < 0.01 were statistically significant

As shown in Supplementary Fig. 1, the proportion of patients with ≥ S2 in the groups of age ≥ 40 years was higher than that in the group of age < 40 years (64.5% vs. 39.9%, *p* < 0.05). Further stratified by ALT levels, in the group of patients age < 40 years, the proportion of ≥ G2 and ≥ S2 in the group with high normal ALT was higher than that in the group with low normal ALT (48.4% vs. 32.1%, 45.3% vs. 30.2%, *p* values were 0.054, 0.073, respectively), but there was no statistical difference. When the patients’ age was ≥ 40 years, it was observed that the proportion of patients with ≥ G2 in the group with high normal ALT was significantly higher than that in the group with low normal ALT (62.5% vs. 14.3%, *p* < 0.05) (Fig. 2). According to different ages and HBV DNA levels, the patients were further analyzed. In the age < 40 years group, patients with HBV DNA ≥ 10^7^ IU/mL had a lower proportion of ≥ S2 and ≥ G2/S2 than those with HBV DNA < 10^7^ IU/mL (36.1% vs. 73.3%, 51.1% vs. 86.7%, *p* < 0.01) (Fig. 2 and Supplementary Table 2).

### Subgroup comparison of different HBV DNA levels

**Fig. 3:**
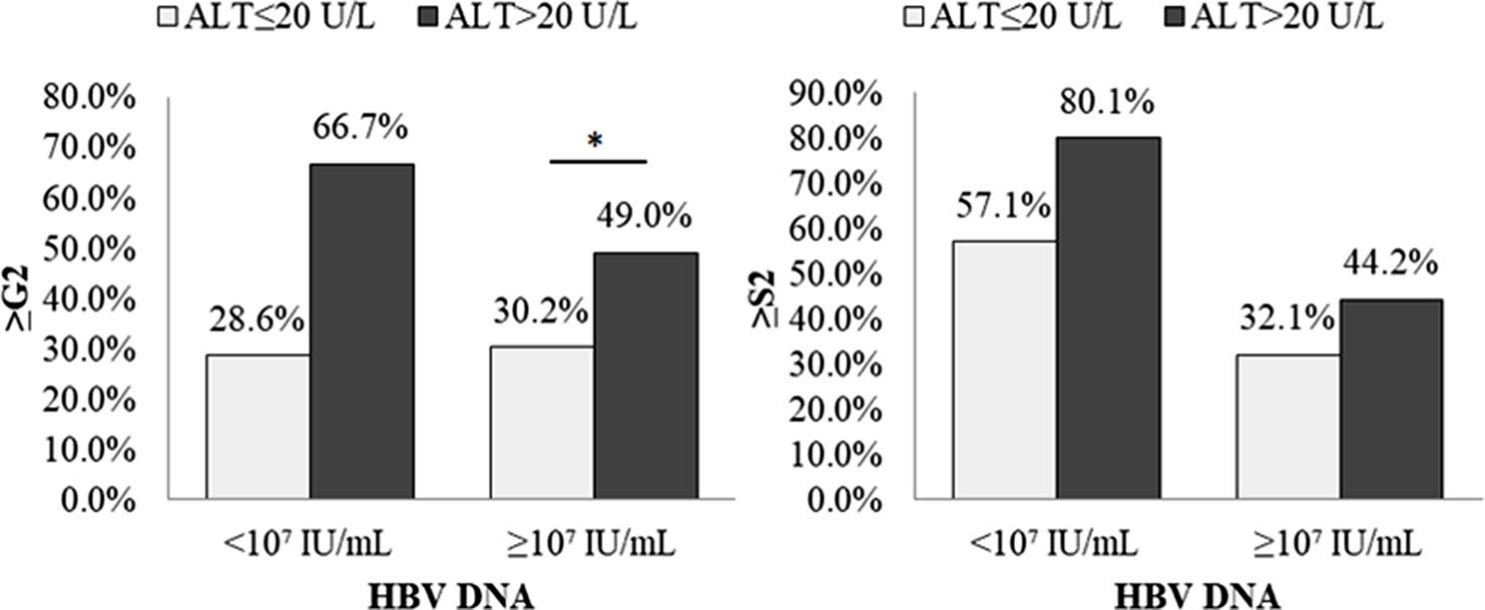
Significant liver histopathology in HBeAg-postive CHB patients in different HBV DNA and ALT groups. Statistical analysis was performed by chi-square test, *p < 0.05, **p < 0.01 were statistically significant

As shown in Supplementary Fig. 2, patients with HBV DNA < 10^7^ IU/mL had a higher proportion of ≥ S2 and ≥ G2/S2 than that in the group with HBV DNA ≥ 10^7^ IU/mL(72.7% vs. 40.1%, *p* < 0.01; 81.8% vs. 54.1%, *p* < 0.05). In addition, 42.7% and 40.1% of patients with HBV DNA ≥ 10^7^ IU/mL had ≥ G2 and ≥ S2, respectively. When comparing different ALT levels, as shown in Fig. 3 and Supplementary Table 2, regardless of HBV DNA < 10^7^ IU/mL or ≥ 10^7^ IU/mL, the high-normal ALT group had a higher proportion of ≥ G2 than the low-normal ALT group, and a significant difference was found in patients with HBV DNA ≥ 10^7^ IU/mL (49.0% vs. 30.2%, *p* = 0.024).

### Predictor forsignificant liver histology

As depicted in Supplementary Table 3, Spearman correlation confirmed a positive correlation between age and liver fibrosis (*r* = 0.262, *p* < 0.01), and HBV DNA levels in HBeAg-positive CHB patients correlated with liver necroinflammation and fibrosis negatively (*r*=-0.181, *p* < 0.05; *r*=-0.217, *p* < 0.01). As shown in Table 2, multivariate logistic regressions were performed to analyze and predict independent risk factors for significant liver histology, showing that ALT > 20 U/L was an independent risk factor for predicting significant liver necroinflammation (OR = 2.855, 95% CI 1.421–5.734, *p* = 0.003), while HBV DNA < 10^7^ IU/mL was an independent risk factor for predicting significant liver fibrosis (OR = 4.173, 95% CI 1.483–11.741, *p* = 0.007).

**Table 2:** Multivariate logistic regression analysis for risk factors predicting significant liver histology in HBeAg-positive CHB patients

	≥G2			≥S2		
	**OR**	**95%CI**	*p* value	**OR**	**95%CI**	*p* value
Age						
< 40 years	1.000			1.000		
≥ 40 years	1.160	0.518–1.447	0.718	2.208	0.954–5.107	0.064
Sex						
Male	1.000					
Female	1.878	0.988–3.570	0.054	1.835	0.957–3.515	0.067
ALT						
≤ 20 U/L	1.000			1.000		
> 20 U/L	2.855	1.421–5.734	0.003	1.958	0.976–3.929	0.059
HBV DNA						
≥ 10^7^ IU/mL	1.000			1.000		
< 10^7^ IU/mL	1.771	0.691–4.538	0.234	4.173	1.483–11.741	0.007

### Incidence of liver cirrhosis and HCC in CHB patients during follow-up

Liver cirrhosis and HCC events occurred during the follow-up period, as shown in Table 3. Follow-up data were available in 36 immune tolerant patients (mean 5.9 years) and 55 indeterminate patients (mean 4.5 years). In this study, during 212 person-years of follow-up, 2 immune tolerant patients developed cirrhosis, with an annual incidence of 0.94%.One immune tolerant patients developed HCC, with an annual incidence of 0.47%. Besides, during 248 person-years of follow-up, 4 indeterminate patients developed cirrhosis, with an annual incidence of 1.6%, and 1 indeterminate patient developed HCC, with an annual incidence of 0.4%, respectively. Meanwhile, all of the immune tolerant patients experienced a phase change during follow up; none of them remained in the immune tolerant phase, 21 indeterminate patients changed to other phase.

**Table 3:** Incidence of cirrhosis and HCC during follow-up n (%)

	Immune tolerance (*n* = 36)	indeterminate (*n* = 55)
**Mean follow-up time (ys)**	5.9	4.5
**Cirrhosis**	2	4
**HCC**	1	1
**Phase Staging**		
**immune tolerance**	7 (19.4)	-
**immune clearance**	5 (13.9)	10 (18.2)
**inactive carrier**	-	4 (7.3)
**reactive**	-	7 (12.7)
**indeterminate**	24 (66.7)	34 (61.8)

## Discussion

It is generally considered that liver biopsy of immune tolerance patients shows no liver fibrosis and mild liver necroinflammation [13], a study showed that the progression of liver injury in immune tolerance patients with a course of more than 5 years was very slow, with only 3 cases (6.3%, 3/48) progressing to fibrosis [14]. Therefore, the main international guidelines recommend that true immune tolerance patients should be closely observed rather than be given antiviral therapy. However, a number of recent studies have found that approximately 1/3 − 1/2 of HBeAg-positive patients with normal ALT have obvious liver histological changes [9, 15–17], indicating that these patients have a high risk of progressing to cirrhosis and HCC. Similarly, in our study, 42.7% and 40.1% of HBeAg-positive patients with normal ALT and HBV DNA ≥ 10^7^ IU/mL had ≥ G2 and ≥ S2, respectively. Our study confirmed that among a noticeable number of HBeAg-positive patients with normal ALT and high HBV DNA levels, significant liver histological changes still exist, suggesting that these patients have disease progression and should be given antiviral therapy. Therefore, it is necessary to look for true immune tolerance patients and to reconsider the patients who meet the antiviral indications and the timing of antiviral treatment among HBeAg-positive CHB patients with normal ALT.

Our study reveals that in HBeAg-positive patients, the proportion of ≥ G2 in the group with high normal ALT was higher than that in the low- normal ALT group (51.3% vs. 30.0%, *p* < 0.01), which was also found in patients aged > 40-year-old and the HBV DNA ≥ 10^7^ IU/mL group. Several studies have presented similar results [18]. Lai et al. cconducted liver biopsy on CHB patients with persistently normal ALT (56% HBeAg positive) and discovered that 46% of patients with high-normal ALT had significant liver necroinflammation or liver fibrosis, which was quite higher than the 20% in the low-normal ALT group [19]. Wong et al. employed transient elastography to examine 254 “immune tolerant” patients, and the proportions of patients over 35 years old with ≥ F2 in the high and low normal ALT groups were 37% and 2%, respectively [20]. These studies demonstrated that patients with high normal ALT had a higher proportion of liver histopathological changes, suggesting that patients with high-normal ALT have an increased risk of disease progression. ALT > 20 U/L was found as a risk factor for predicting significant liver necroinflammation independently through logistic regression analysis. Therefore, we recommend performing liver biopsy and/or implementing noninvasive approaches for patients with high normal ALT to determine whether antiviral therapy is necessary. On the other hand, previous study have shown that male patients were associated with adverse progression of CHB, but our study demonstrated that it did not emerge as significant factor in the multivariate analyses. To identify the reason of inconsistency, we compared the proportion of male and female patients who had significant liver histopathology with ALT > 20 IU/L (supplementary Table 5), and it showed that a higher proportion of female patients have significant liver histology than male patients (72.3% vs. 55.6%, *p* = 0.065), although these results were not statistically significant. The explanation of this result was that ALT > 20 IU/L may be a normal level for male but abnormal for female. Consequently, the relationship between gender and liver histopathology needs to be further investigation, and gender factors should also be taken into account when evaluating the ULN level of ALT.

Patient age is also a key factor in deciding the initiation of antiviral therapy. Currently, the age cutoff value for immune tolerance has not been clearly delineated. The AASLD guidelines point out that patients aged > 40 years are more prone to have significant liver histological disease, and antiviral therapy is recommended for immune tolerance patients aged > 40 years [3]. The EASL guidelines recommend that patients with an ALT of 41–80 U/L should be evaluated for liver histology via transient elastography and/or liver biopsy and antiviral therapy should be contemplated for patients over 30 years old, even if liver fibrosis is not assessed [4]. A study involving 253 CHB patients demonstrated that when ALT was normal (ULN:30 U/L for males; 19 U/L for females), the proportions of ≥ F2 in patients aged over 40 and under 40 years were 42% and 30%, respectively [21]. In our study, it was noted that the ≥ 40-year-old group exhibited a higher proportion of ≥ S2 than the < 40-year-old group (64.5% vs. 39.9%, *p* < 0.05). Furthermore, this study revealed that 39.9% of patients with normal ALT and an age < 40 years have significant liver fibrosis, which indicates that more rigorous monitoring of liver fibrosis is also necessary for patients aged < 40 years, and more data are needed in the future to investigate the relationship between age and the immune tolerance phase.

The interaction between HBV DNA and liver histological changeshas still not been completely clarified. Previous studies have found that HBV DNA levels of HBeAg-negative patient are positively correlated with liver injuries [22, 23], while Bai et al. found that in HBeAg-positive patients, there was no significant relation between them [24]. In this study, among HBeAg-positive patients with normal ALT, HBV DNA levels are negatively correlated with liver fibrosis (*r*=-0.217, *p* = 0.004), (Supplementary Figs. 3 and 4), which is in conformity with the results of a previous study of HBeAg-positive patients(*r*=-0.491, *p* < 0.001) [25]. We also found that the proportion of ≥ G2/S2 in the HBV DNA < 10^7^ IU/mL group was higher than that in the group with HBV DNA ≥ 10^7^ IU/mL (81.8% vs. 54.1%, *p* < 0.05). Similar results were observed in patients aged < 40 years or ≥ 30 years (Supplementary Table 4). However, when the age was ≥ 40 years, there was no difference in the proportion of significant liver histopathology between them, which further indicates that when the age is higher than 40 years, most patients are no longer in the true immune tolerance phase, and antiviral therapy is needed. In addition, research on the relationship between HBV DNA levels and HCC had shown that patients with persistently high HBV DNA levels (> 10^7^ IU/mL) had a significantly lower risk of HCC than patients with HBV DNA levels between 10^6^~10^7^ IU/mL [26]. Moreover, Lee et al. discovered that the cutoff of HBV DNA > 10^7^ IU/mL can be used to determine the immune tolerance phase [27]. In our study, it can be seen that HBV DNA < 10^7^ IU/mL is a risk factor for independently predicting liver fibrosis ≥ S2, which indicats that patients with HBV DNA > 10^7^ IU/mL are more likely to be truly immune tolerant. In the future, more research is demanded to figure out the cutoff value of HBV DNA during immune tolerance. Thus, it is important to monitor “immune tolerant” patients with older age, higher normal ALT and lower HBV DNA levels closely who might have a high risk of advanced disease.

Recently, a fiery discussion is focused on whether to expand antiviral treatment indicators in CHB patients, especially in immune-tolerant patients [28, 29]. In our study, a higher proportion of indeterminate patients were reported, therefore, it is necessary to further explore how to determine the true immune tolerant patients. In fact, there have been studies consistent with our research. A Korean study conducted by Yoo et al. reported that nearly 70% of patients were not in the true immune-tolerant phase [30]. Another Chinese research also showed that over 30% of immune tolerant CHB patients had significant liver fibrosis [31]. Both of them have demonstrated that lower HBV DNA levels was associated with significant liver injuries. In a European setting, Göbel T et al. demonstrated that patients with CHB infection and normal ALT levels had significant liver fibrosis(36%) or necroinflammation(27%) [21]. At the same time, the indeterminate patients have a phase change tendency and may develop into liver cirrhosis and HCC during follow up. Chinese guidelines (2022 versions) suggest deciding whether antiviral therapy needs to be initiated based on a comprehensively disease progression by assessing serum HBV DNA, ALT level, and liver disease severity, combined with factors such as age, family history, and concomitant diseases [32]. It is expected that more research will be carried out on the effectiveness of antiviral therapy for patients in the immune tolerance phase, so as to better manage the patients in this phase individually.

Our study focused on the hot issues regarding the management of ALT-normal CHB patients; however, there were several limitations. First, the sample size was not large enough. The patients in this study were collected from one provincial hospital. Second, we lack the data on obesity and diabetes, which are potential cofounders of liver histopathology. Third, it was also limited by the inadequate data of follow-up and we didn’t have data on HBV genotypes of the study population, which might better illustrate the dynamic changes of the long-term prognosis. Therefore, we could enroll more cases from different region in the future to better explore the prognosis of ALT-normal CHB patients.

In summary, our research showed that (57.5%) significant liver histological changes were revealed in HBeAg-positive CHB patients with normal ALT, and 81.8% of patients with HBV DNA < 10^7^ IU/mL had significant liver histopathology. Based on our finding, liver biopsy or noninvasive approaches are strongly recommended for patients with high-normal ALT or with HBV DNA < 10^7^ IU/mL to evaluate the degree of liver histological changes and to recommend antiviral therapy for patients with significant liver histopathology. Future studies should be carried out to explore the need for antiviral therapy and to individualize the management of those indeterminate patients.

### Electronic supplementary material

Below is the link to the electronic supplementary material.

Supplementary Material 1

Supplementary Material 2

## Data Availability

The data that support the findings of this study are available on request from the corresponding author.
