# Supplementary material for: Significant liver histological change is common in HBeAg-positive chronic hepatitis B with normal ALT
Source: BMC Infect Dis. 2024 Jul 23;24:723. doi: 10.1186/s12879-024-09617-1 (PMC11264461; doi:10.1186/s12879-024-09617-1)
Supplement: Supplementary file 1 — Supplementary Material 1 [file 12879_2024_9617_MOESM1_ESM.docx]

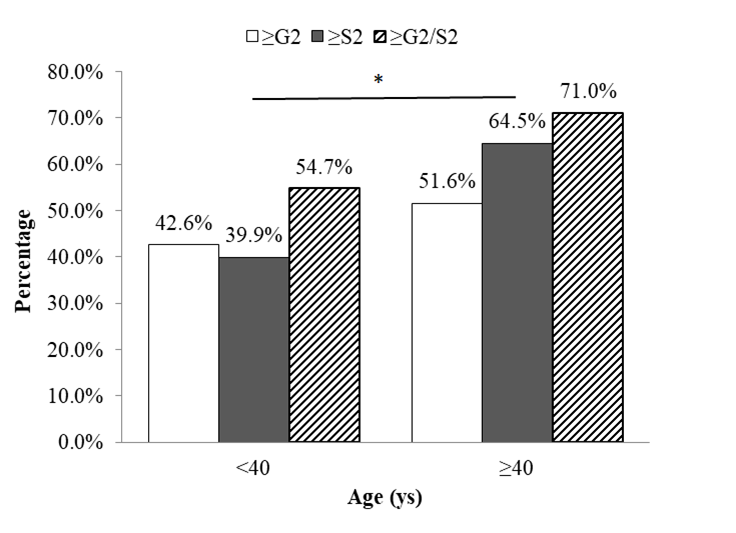


**Supplementary figure 1.** Significant liver histopathology in HBeAg-postive CHB patients in different age groups.**p*<0.05


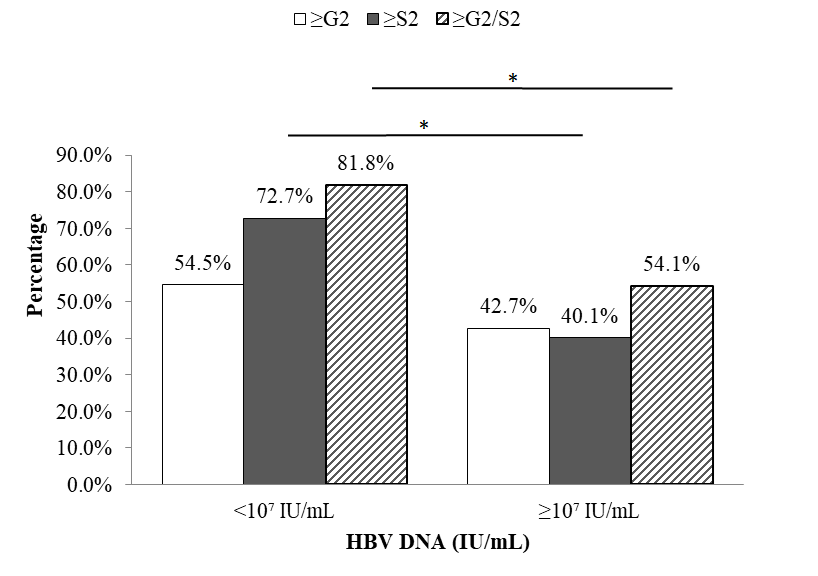


**Supplementary figure 2.** Significant liver histopathology in HBeAg-postive CHB patients in different HBV DNA groups. **p*<0.05


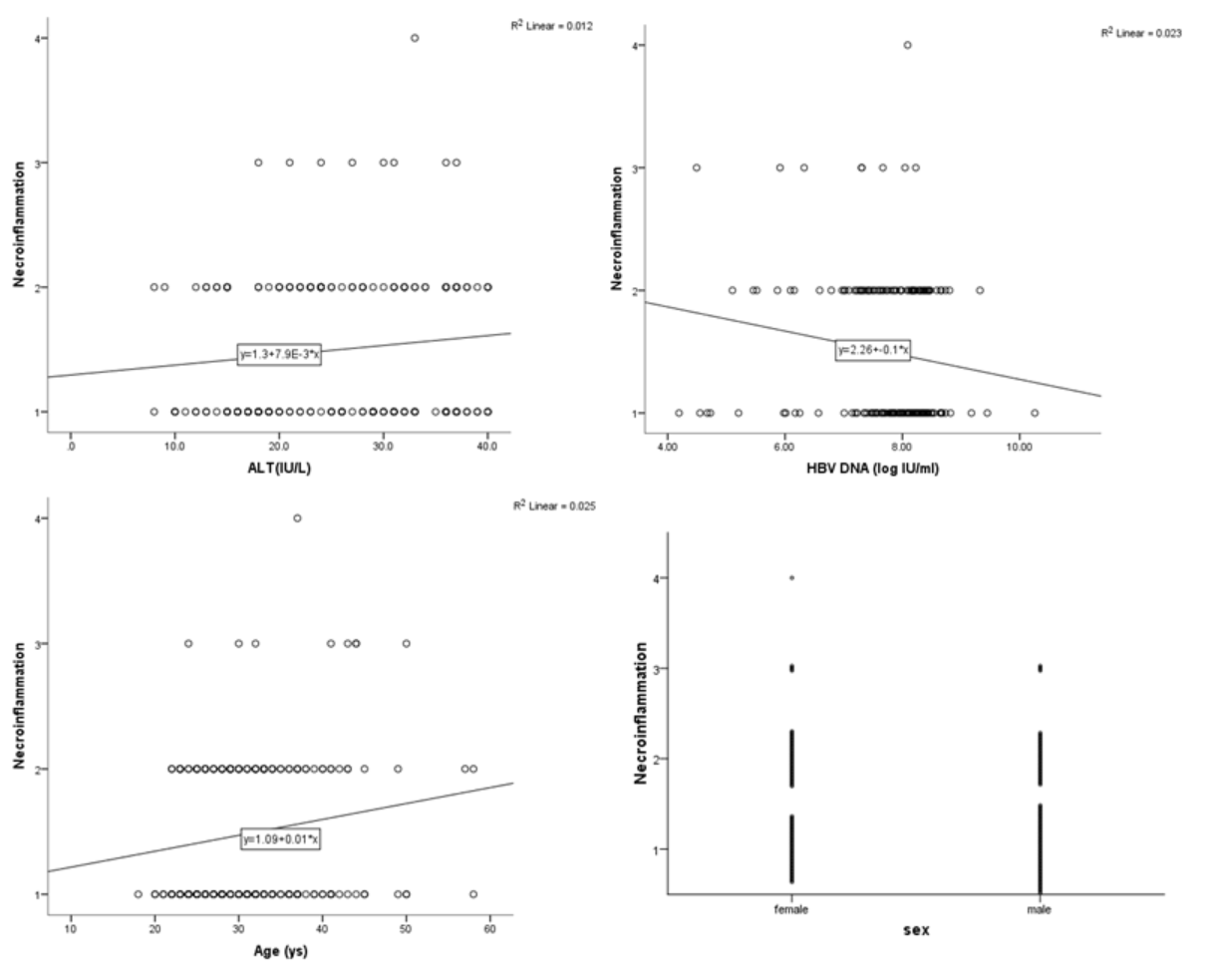


**Supplementary figure 3.** Dot-plot of correlation between liver necroinflammation and clinical factors in HBeAg-positive CHB patients

**
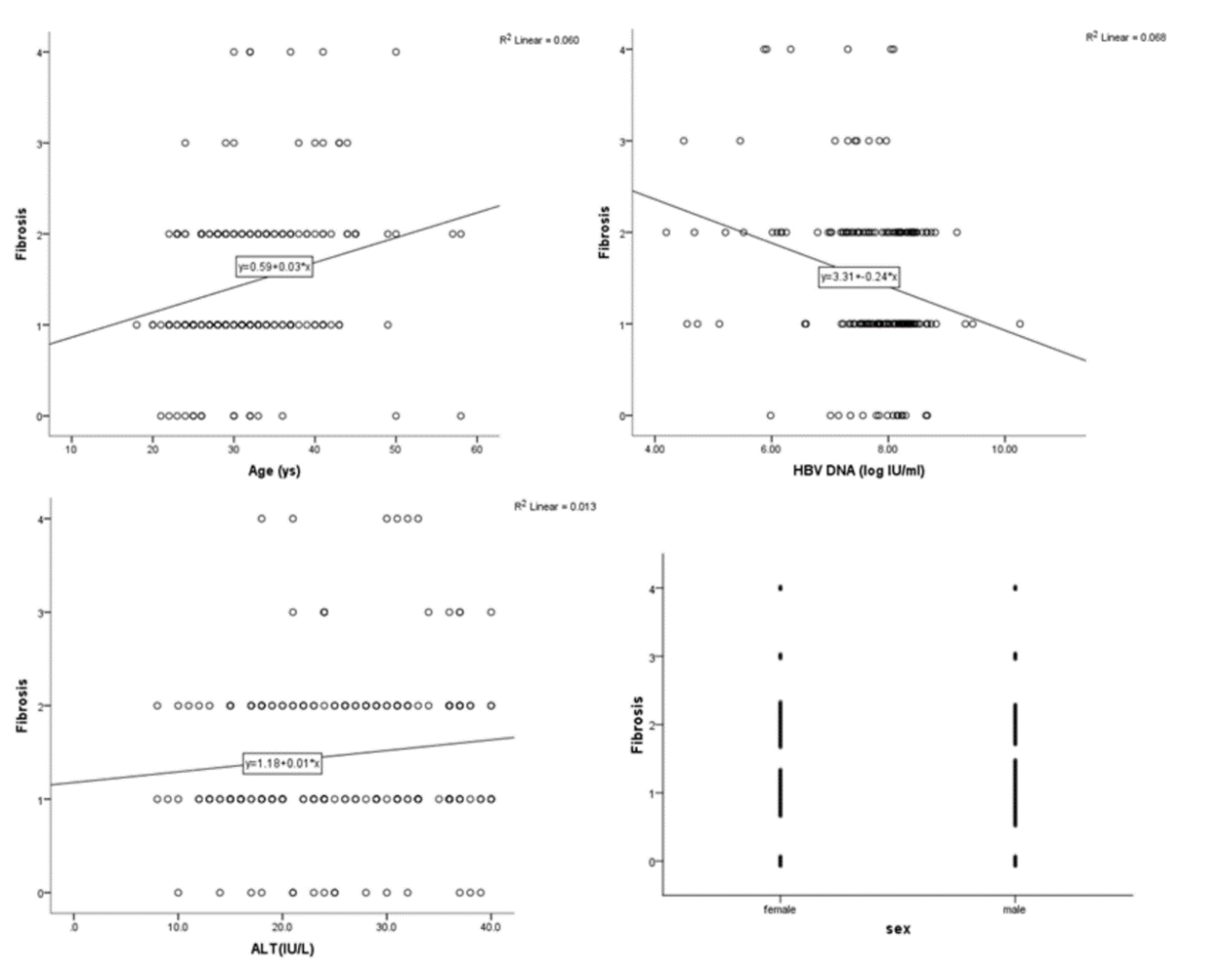
**

**Supplementary figure 4.** Dot-plot of correlation between liver fibrosis and clinical factors in HBeAg-positive CHB patients
