# Supplementary material for: Significant liver histological change is common in HBeAg-positive chronic hepatitis B with normal ALT
Source: BMC Infect Dis. 2024 Jul 23;24:723. doi: 10.1186/s12879-024-09617-1 (PMC11264461; doi:10.1186/s12879-024-09617-1)
Supplement: Supplementary file 2 — Supplementary Material 2 [file 12879_2024_9617_MOESM2_ESM.docx]

**Supplementary Table 1** Distribution of liver histology in different age and HBV DNA groups in HBeAg-positive CHB patients (%)

| **Histological**  **grading** | **<40 years**  **(148)** | | | *p* value | **≥40 years**  **(31)** | | *p* value |
| --- | --- | --- | --- | --- | --- | --- | --- |
|  | **<10^7^ IU/mL**  **(15)** | | **≥10^7^ IU/mL**  **(133)** |  | **<10^7^ IU/mL**  **(7)** | **≥10^7^ IU/mL**  **(24)** |  |
| G1 | 6 (40.0) | 79 (59.4) | |  | 4 (57.1) | 11 (45.8) |  |
| ≥G2 | 9 (60.0) | 54 (40.6) | | 0.150 | 3 (42.9) | 13 (54.2) | 0.598 |
| S0 | 0 (0) | 14 (10.5) | |  | 1 (14.3) | 1 (4.2) |  |
| S1 | 4 (26.7) | 71 (53.4) | |  | 1 (14.3) | 8 (33.3) |  |
| ≥S2 | 11 (73.3) | 48 (36.1) | | 0.005 | 5 (71.4) | 15 (62.5) | 0.664 |
| ≥G2/S2 | 13 (86.7) | 68 (51.1) | | 0.009 | 5 (71.4) | 17 (70.8) | 0.976 |

**Supplementary Table 2** Distribution of liver histology in different HBV DNA and ALT groups in HBeAg-positive CHB patients (%)

| **Histological**  **grading** | **<10^7^IU/mL**  **(22)** | | | ***p* value** | **≥10^7^ IU/mL**  **(157)** | | ***p* value** |
| --- | --- | --- | --- | --- | --- | --- | --- |
|  | **ALT≤20 U/L**  **(7)** | | **ALT>20 U/L**  **(15)** |  | **ALT≤20 U/L**  **(53)** | **ALT>20 U/L**  **(104)** |  |
| G1 | 5 (71.4) | 5 (33.3) | |  | 37 (69.8) | 53 (51.0) |  |
| ≥G2 | 2 (28.6) | 10 (66.7) | | 0.095 | 16 (30.2) | 51 (49.0) | 0.024 |
| S0 | 0 (0) | 1 (6.7) | |  | 4 (7.5) | 11 (10.6) |  |
| S1 | 3 (26.7) | 2 (13.3) | |  | 32 (60.4) | 47 (45.2) |  |
| ≥S2 | 4 (57.1) | 12 (80.0) | | 0.262 | 17 (32.1) | 46 (44.2) | 0.142 |
| ≥G2/S2 | 5 (71.4) | 13 (86.7) | | 0.388 | 24 (45.3) | 61 (58.7) | 0.112 |

**Supplementary Table 3** Correlation between liver histopathology and clinical factors in HBeAg-positive CHB patients

|  | **Necroinflammation** | | **Fibrosis** | |
| --- | --- | --- | --- | --- |
|  | Correlation coefficient | *p* value | Correlation coefficient | *p* value |
| ALT | 0.106 | 0.159 | 0.109 | 0.145 |
| Age | 0.133 | 0.076 | 0.262 | 0.000 |
| Sex | 0.091 | 0.227 | 0.063 | 0.406 |
| HBV DNA | -0.181 | 0.016 | -0.217 | 0.004 |

**Supplementary Table 4** Distribution of liver histology in different age and HBV DNA groups (%) in HBeAg-positive CHB patients

| **Histological**  **grading** | **<30 years**  **(75)** | | | ***p* value** | **≥30 years**  **(104)** | | ***p* value** |
| --- | --- | --- | --- | --- | --- | --- | --- |
|  | **<10^7^ IU/mL**  **(4)** | | **≥10^7^ IU/mL**  **(71)** |  | **<10^7^ IU/mL**  **(18)** | **≥10^7^ IU/mL**  **(86)** |  |
| G1 | 2 (50.0) | 43 (60.6) | |  | 8 (44.4) | 47 (54.7) |  |
| ≥G2 | 2 (50.0) | 28 (39.4) | | 0.675 | 10 (55.6) | 39 (45.3) | 0.430 |
| S0 | 0 (0) | 8 (11.3) | |  | 1 (5.6) | 7 (8.1) |  |
| S1 | 1 (25.0) | 40 (56.3) | |  | 4 (22.2) | 39 (45.3) |  |
| ≥S2 | 3 (75.0) | 23 (32.4) | | 0.081 | 13 (72.2) | 40 (46.5) | 0.047 |
| ≥G2/S2 | 3 (75.0) | 35 (49.3) | | 0.317 | 15 (83.3) | 50 (58.1) | 0.045 |

**Supplementary Table 5** Distribution of significant liver histology in different sex group in patients with ALT>20 IU/L (%)

| Liver histology | Male  (n=72) | Female  (n=47) | *p* value |
| --- | --- | --- | --- |
| ≥G2/S2 | 40(55.6) | 34 (72.3) | 0.065 |
